# Conjunction of ovarian dermoid cyst in a patient with Pituitary Micro Adenoma: A Case Report

**DOI:** 10.31729/jnma.9122

**Published:** 2025-06-30

**Authors:** Saroj Babu Aryal, Shasank Chitrakar, Akriti Panthi

**Affiliations:** 1 Department of Clinical Biochemistry, Maharajgunj Medical Campus, Tribhuwan University Medical Campus, Maharajgunj, Kathmandu, Nepal; 2 Department of General, Physician and Emergency Medicine, Maharajgunj Medical Campus, Tribhuwan University Medical Campus Maharajgunj, Kathmandu, Nepal; 3 Department of Clinical Pathology, Maharajgunj Medical Campus, Tribhuwan University Medical Campus, Maharajgunj, Kathmandu, Nepal

**Keywords:** *adenoma*, *dermoid*, *gynecological*, *ovarian*, *prolactin*

## Abstract

Pituitary adenomas are common intracranial tumors, and ovarian dermoids are frequently encountered benign gynecological lesions. While both conditions are individually prevalent, their co-existence is rare and infrequently reported. This unusual combination may pose diagnostic challenges and warrants further exploration to determine any potential underlying link. We present a case of a 42-year-old Hindu female who was diagnosed case of a pituitary adenoma with a leftsided ovarian dermoid cyst resulting in a gynecological and hormonal imbalance. The diagnosis was confirmed by subsequent related investigations and the patient has been getting symptomatic treatment. This case underscores the importance of a comprehensive differential diagnosis in patients presenting with related symptoms and highlights the value of a multidisciplinary approach for timely diagnosis, effective management, and improved patient outcomes. Despite advancements, the relationship between elevated prolactin levels and the pathophysiology of ovarian dermoid cysts remains unclear. The synchronous occurrence of these two conditions is rare and continues to pose a diagnostic and therapeutic challenge for clinicians.

## INTRODUCTION

Mature cystic teratomas are the most common benign ovarian germ cell tumours, usually found in women in their second and third decades.^1,2^ Histologically, they contain well-differentiated tissue from all three germ layers and are often asymptomatic, discovered incidentally during imaging or surgery. Prolactin, secreted by pituitary lactotroph cells, regulates reproductive function; elevated levels in nonpregnant women can cause menstrual irregularities, infertility, and galactorrhoea.^3^ A common cause of hyperprolactinaemia is a functioning pituitary adenoma. Serum prolactin levels above 20 ng/mL are abnormal in reproductive-aged women.^4^ This report presents a rare case of co-existing ovarian dermoid cyst and pituitary microadenoma, managed with cabergoline.

## CASE PRESENTATION

A 42-year-old non-smoker, non-alcoholic Hindu female with no prior medical history presented to Tribhuvan University Teaching Hospital outpatient department with bilateral breast pain and milky discharge lasting 18 months. The breast pain was insidious, continuous, mild to moderate, and constricting without radiation. Additional symptoms included headache, diplopia, blurred vision, skin darkening for one month, and mild lower abdominal pain with heaviness. There was no history of nausea, vomiting, trauma, or neurological deficits. Physical examination revealed no breast masses or axillary lymphadenopathy, and systemic examination was unremarkable.

**Figure 1:**
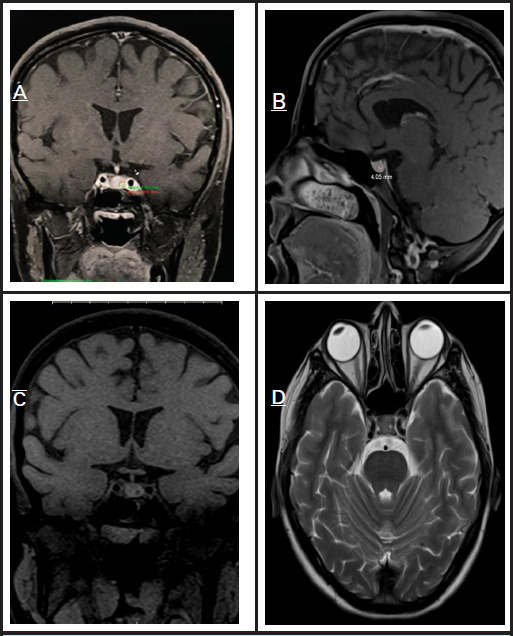
MRI images of the pituitary gland demonstrate a single nodule consistent with a pituitary adenoma located on the left-posterior aspect. (A) Coronal view showing the nodule on the left- posterior side of the pituitary gland. (B) Sagittal view revealing the same lesion measuring approximately 4 x 4 mm. (C) Coronal view confirming the localization of the nodule. (D) Axial view further supports the presence of the lesion on the left-posterior aspect.

Laboratory tests including LFT, RFT, CBC, and pregnancy test were normal, but serum prolactin was markedly elevated at 2928 uIU/ml (normal 66-500 uIU/ml). Breast and axillary ultrasound were normal, while abdominal ultrasound showed a left ovarian dermoid cyst measuring 3.5 x 3.4 cm. Brain MRI identified a 4 x 4 mm hypo-enhancing lesion on the posterior left pituitary gland consistent with a pituitary microadenoma (Fig. 1).

Based on clinical, biochemical, and imaging findings, a diagnosis of pituitary microadenoma with a left ovarian dermoid cyst was made. The patient was treated with cabergoline 0.5 mg twice weekly for one year, resulting in significant improvement of breast tenderness and galactorrhea, with ongoing follow-up advised.

## DISCUSSION

A dermoid cyst is a benign tumor containing cystic structures filled with tissues typically found in the outer skin layers, such as sweat and oil glands, and may also include hair and teeth. These cysts can develop anywhere in the body but are most commonly located in the ovaries, testes, skin of the head, neck, face, lower back, or central nervous system. Classified as a type of germ cell tumor, dermoid cysts are also known as mature teratomas.^5^ The development of ovarian dermoid cysts is associated with the hedgehog signaling pathway, which plays a key role in their molecular pathogenesis.^6^

Prolactin is a hormone primarily synthesized and secreted by lactotroph cells of the pituitary gland and functions through the JAK-STAT kinase signaling pathway. In addition to its pituitary origin, prolactin is also produced at extra-pituitary sites such as the placenta, uterus, mammary glands, and T lymphocytes, reflecting its diverse physiological roles beyond lactation.^7^

Currently, no studies have established a direct interconnection between the Hedgehog signaling pathway and the JAK-STAT kinase pathway. Pituitary adenomas are typically benign tumors that manifest symptoms through either compression of adjacent structures or hormone secretion abnormalities. Treatment strategies, whether surgical or medical, depend on the tumor subtype and extent of local invasion. The pathogenesis of pituitary adenomas involves deregulation of molecular pathways, angiogenesis, and cell cycle disruption. Key signaling pathways implicated in their development include Notch, Wnt, and Hedgehog, all of which are crucial during early pituitary organogenesis. These pathways play an essential role in the formation of various pituitary cell types, including somatotrophs, lactotrophs, thyrotrophs, and corticotrophs, thereby contributing to tumorigenesis when dysregulated.^6^

Prolactinomas account for approximately 40% to 57% of all pituitary adenomas, making them the most common subtype, followed by nonfunctioning adenomas (28% to 37%), growth hormone-secreting adenomas (11% to 13%), and ACTH-secreting adenomas (1% to 2%). A hallmark feature of prolactinomas is hormone hypersecretion, particularly hyperprolactinemia. Neurologic symptoms such as headaches and visual disturbances are frequently observed in patients with pituitary adenomas, often resulting from the tumor's pressure on surrounding structures, including the optic chiasm.^8^

Pituitary adenomas can present with a range of signs and symptoms, including galactorrhea, decreased libido, infertility, gynecomastia, impotence in men, and menstrual irregularities such as oligomenorrhea or amenorrhea. Headaches are a common complaint, typically resulting from stretching of the dural sheath, though they are non-specific and do not necessarily correlate with tumor size. As the tumor enlarges, it may compress the optic chiasm superiorly, leading to visual field deficits —most commonly bitemporal hemianopia. In cases of more severe compression, visual acuity may also decline. Unlike headaches, visual disturbances generally correlate with the size of the tumor.^8^

A previously published case report described a 34-year- old female who presented with a 1.5-year history of secondary galactorrhea and amenorrhea, with a serum prolactin level of 151.89 ng/mL. Despite treatment with dopamine agonists, including cabergoline and bromocriptine, her prolactin levels remained elevated. Pituitary imaging showed no abnormalities, prompting further investigation for an ectopic source of prolactin. Imaging revealed a heterogeneous mass originating from the uterus, and octreotide scintigraphy confirmed uptake consistent with the uterine mass. The patient subsequently underwent a total abdominal hysterectomy, and her prolactin levels dropped significantly to 0.4 ng/mL the following day. This case suggests a rare association between uterine myoma and hyperprolactinemia, particularly when resistant to conventional medical therapy.^9^

A previously reported case described a 29-year-old nulliparous woman who presented with intermittent left lower quadrant pain, galactorrhea, night sweats, hot flushes, and oligomenorrhea. She had a history of regular menstrual cycles from menarche at age 15 until age 22, after which she experienced increasing irregularity and eventual amenorrhea. Laboratory evaluation revealed a markedly elevated serum prolactin level of 930.8 ng/mL. Pelvic ultrasound identified a complex left adnexal mass, suggestive of either a hemorrhagic cyst or a cystic teratoma. However, MRI imaging of the brain showed no evidence of a pituitary mass. This case highlights the importance of considering extrapituitary sources of hyperprolactinemia, particularly when pituitary imaging is normal.^10^

Another case report described a patient initially diagnosed with a pituitary mass, which was managed surgically but without success in resolving symptoms. Subsequently, medical therapy was initiated, leading to symptomatic relief. However, several years later, the patient developed ectopic prolactin production originating from ovarian tissue. This case emphasizes the potential for ectopic prolactin secretion even after primary pituitary pathology has been addressed and highlights the need for long-term monitoring and consideration of alternative sources in cases of recurrent or persistent hyperprolactinemia.^11^

## CONCLUSIONS

In cases of significantly elevated prolactin levels accompanied by a pituitary adenoma, there may also be an underlying ovarian dermoid, warranting thorough pelvic evaluation. Pelvic examination and imaging should be included in the diagnostic workup for hyperprolactinemia in women to identify potential ectopic sources of prolactin production. While hyperprolactinemia is commonly attributed to pituitary lactotroph adenomas, it can also result from functioning lactotroph-like cells present within ovarian dermoids. Recognizing this possibility is essential for accurate diagnosis and effective management.

## References

[1] Zalel Y, Piura B, Elchalal U, Czernobilsky B, Antebi S, Dgani R (1996). Diagnosis and management of malignant germ cell ovarian tumors in young females.. Int J Gynecol Obstet..

[2] Killackey MA, Neuwirth RS (1988). Evaluation and management of the pelvic mass: a review of 540 cases.. Obstet Gynecol..

[3] Melmed S, Casanueva FF, Hoffman AR, Kleinberg DL, Montori VM, Schlechte JA, Wass JAH (2011). Diagnosis and treatment of hyperprolactinemia: An Endocrine Society Clinical Practice Guideline.. J Clin Endocrinol Metab..

[4] Molitch ME (2007). Long-Term Management of Prolactinomas.. J Clin Endocrinol Metab..

[5] NCI Dictionary of Cancer Terms. (2011). Definition of dermoid cyst..

[6] M S, DC, V M, P O, S O, I W (2012). The Hedgehog signaling pathway in ovarian teratoma is stimulated by Sonic Hedgehog which induces internalization of Patched.. Int J Oncol..

[7] Bachelot A, Binart N (2007). Reproductive role of prolactin.. Reproduction..

[8] Lake MG (2013). Pituitary Adenomas: An Overview.. Am Fam Physician..

[9] Simsir IY, Kocabas GU, Sahin SB, Erdogan M, Cetinkalp S, Saygili F (2012). A case of an ectopic prolactinoma.. Gynecol Endocrinol..

[10] Elms AF, Carlan SJ, Rich AE, Cerezo L (2012). Ovarian tumor-derived ectopic hyperprolactinemia.. Pituitary..

[11] Kallenberg GA (1990). Ectopic Hyperprolactinemia Resulting From an Ovarian Teratoma.. JAMA..

